# Consumption of a High-Protein Meal Replacement Leads to Higher Fat Oxidation, Suppression of Hunger, and Improved Metabolic Profile After an Exercise Session

**DOI:** 10.3390/nu13010155

**Published:** 2021-01-05

**Authors:** Camila L. P. Oliveira, Normand G. Boulé, Aloys Berg, Arya M. Sharma, Sarah A. Elliott, Mario Siervo, Sunita Ghosh, Carla M. Prado

**Affiliations:** 1 Human Nutrition Research Unit, Department of Agricultural, Food & Nutritional Science, University of Alberta, Edmonton, AB T6G 2E1, Canada; camila@ualberta.ca (C.L.P.O.); se2@ualberta.ca (S.A.E.); 2 Alberta Diabetes Institute, University of Alberta, Edmonton, AB T6G 2E1, Canada; 3 Faculty of Kinesiology, Sport, and Recreation, University of Alberta, Edmonton, AB T6G 2H9, Canada; 4 Faculty of Medicine, University of Freiburg, 79110 Freiburg, Germany; berg.aloys@web.de; 5 Division of Endocrinology & Metabolism, Department of Medicine, University of Alberta, Edmonton, AB T6G 2G3, Canada; amsharm@ualberta.ca; 6 Alberta Research Centre for Health Evidence, Department of Pediatrics, University of Alberta, Edmonton, AB T6G 1C9, Canada; 7 School of Life Sciences, Division of Physiology, Pharmacology and Neuroscience, University of Nottingham, Nottingham NG7 2UH, England, UK; mario.siervo@nottingham.ac.uk; 8 Department of Medical Oncology, University of Alberta, Edmonton, AB T6G 2R7, Canada; sunita.ghosh@albertahealthservices.ca

**Keywords:** meal replacement, protein, exercise, energy metabolism, appetite

## Abstract

The aim of this study was to compare the impact of a high-protein meal replacement (HP-MR) versus a control (CON) breakfast on exercise metabolism. In this acute, randomized controlled, cross-over study, participants were allocated into two isocaloric arms: (a) HP-MR: 30% carbohydrate, 43% protein, and 27% fat; (b) CON: 55% carbohydrate, 15% protein, and 30% fat. Following breakfast, participants performed a moderate-intensity aerobic exercise while inside a whole-body calorimetry unit. Energy expenditure, macronutrient oxidation, appetite sensations, and metabolic blood markers were assessed. Forty-three healthy, normal-weight adults (24 males) participated. Compared to the CON breakfast, the HP-MR produced higher fat oxidation (1.07 ± 0.33 g/session; *p* = 0.003) and lower carbohydrate oxidation (−2.32 ± 0.98 g/session; *p* = 0.023) and respiratory exchange ratio (−0.01 ± 0.00; *p* = 0.003) during exercise. After exercise, increases in hunger were lower during the HP-MR condition. Changes in blood markers from the fasting state to post-exercise during the HP-MR condition were greater for insulin, low-density lipoprotein cholesterol, peptide tyrosine-tyrosine, and gluca-gon-like peptide 1, and lower for triglyceride and glycerol. Our primary findings were that a HP-MR produced higher fat oxidation during the exercise session, suppression of hunger, and improved metabolic profile after it.

## 1. Introduction

Maintenance of a healthy body weight is essential to decrease morbidity and mortality associated with excess body weight [1,2]. Considering the negative impact of excessive body fat accumulation on individual’s health and on the public health system [2,3], substantial effort has been given to develop guidelines for its prevention and treatment [4]. Some of the factors influencing weight management include the food environment, physical activity, and environmental/behavioral factors [5]. Lifestyle modifications that induce an energy deficit, such as diet and physical activity, are considered the cornerstone of weight management [6].

Diet and physical activity are key players in the “intake” and “expenditure” sides of the energy balance equation [7]. Although the balance concept seems uncomplicated, its regulation is highly complex and influenced not only by energy intake and energy expenditure (EE), but also by physiologic and behavioral factors, such as age, hormones, and appetite sensations [8]. In fact, there is a 1–2% decline in basal metabolic rate per decade of life [9]. Therefore, the “eat less” and “exercise more” solution for weight management is not a simple one. Although caloric restriction and exercise can help with weight loss [10], the long-term weight maintenance can be challenging, and most individuals usually regain their body weight [11]. Part of this response seems to be associated with compensatory adjustments to diet- and exercise-induced perturbations in the energy balance equation, including a decrease in EE, upregulation in appetite and orexigenic hormones (e.g., ghrelin), and a decrease in anorexigenic hormones (e.g., leptin, peptide tyrosine tyrosine [PYY], and glucagon-like peptide 1 [GLP-1]) [12,13], which seem to be regulated differently in females and males [14]. Interestingly, males appear to experience greater exercise-induced weight loss than females [15], who seem to demonstrate higher compensatory responses to exercise in order to preserve body fat stores and reproductive function [16].

Numerous dietary strategies exist and are continuously being developed in an attempt to induce a state of negative energy balance [17]. Among those, weight loss strategies based on meal replacements [18,19,20] and a higher protein intake [21] have been a topic of investigation. A recent systematic review and meta-analysis showed that weight loss was greater after one year of intervention when meal replacements were incorporated in the diets, compared to dietary advice or diet plans [19]. A higher protein intake appears to increase fat oxidation, EE, and spare lean mass during weight loss [22,23]. Moreover, it exerts a stronger satiating effect and appears to decrease energy intake under ad libitum conditions [22]. Although these dietary strategies are gaining popularity worldwide [17], little is known about the effects on the mechanisms involved in body weight regulation of females and males, especially when associated with exercise. It is important to study the physiological impact of these strategies in a healthy, normal-weight population without the confounding effects of obesity and other comorbidities.

We investigated the effects of the consumption of a high-protein meal replacement (HP-MR) versus a control (CON) breakfast (North American) preceding an acute bout of moderate-intensity exercise on selected components of exercise energy metabolism, appetite sensations, and metabolic blood markers in healthy, normal-weight young adults of both sexes. We hypothesized that compared to a typical North American breakfast, participants consuming an isocaloric HP-MR prior to a moderate-intensity aerobic exercise session would present with an energy metabolism profile favoring increased EE and fat oxidation during exercise. Moreover, they would present with an improved metabolic profile and decreased appetite that would reflect an increase in blood levels of anorexigenic hormones and decreased levels of the orexigenic hormone ghrelin following the exercise session.

## 2. Materials and Methods

### 2.1. Study Design and Participant Details

This randomized, controlled, cross-over study was a planned secondary analysis of a 32-h intervention trial and conducted separately in females and males at the University of Alberta (Edmonton, AB, Canada), fully described elsewhere [24]. The results from the primary analysis have also been published [25]. Study protocols were approved by the University of Alberta Ethics Board (Pro00066006 and Pro00083005 approved on 5 October 2016 and on 9 July 2018, respectively) and registered in ClinicalTrials.gov (NCT02811276 and NCT03565510). Both protocols complied with the standards as set out in the Canadian Tri-Council Policy statement on the use of human participants in research. Before study commencement, all participants provided written informed consent.

Eligible individuals were healthy females and males between 18 and 35 years old with a body mass index (BMI) of 18.5 to 24.9 kg/m^2^. Potential participants were excluded if they had any diagnosed acute and/or chronic disease, claustrophobia, dietary restrictions, recent exposure to tests involving radiation, were using medications and/or nutritional supplements that could affect energy metabolism or body composition, and were performing >1 h/day or >7 h/week of exercise. Females with an irregular menstrual cycle, pregnant, or lactating were also excluded.

### 2.2. Experimental Protocol

**Figure 1:**
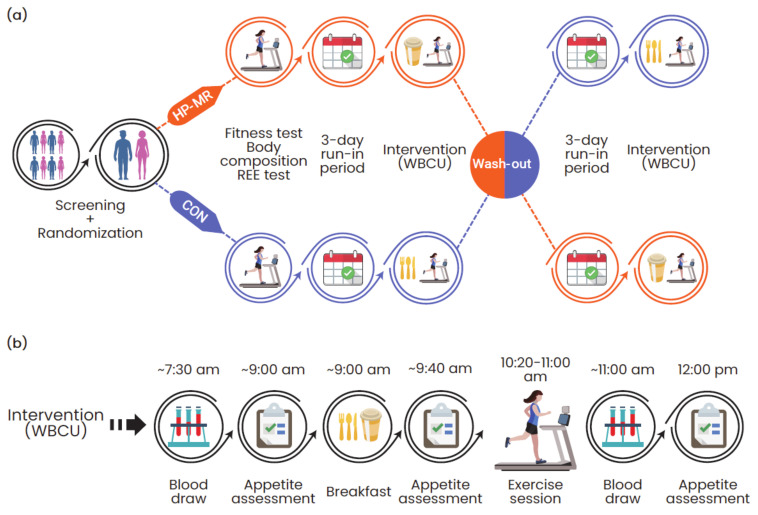
(**a**) Overview of the experimental protocol and (**b**) activities performed during each condition. Abbreviations: CON: control; HP-MR: high-protein meal replacement; REE: resting energy expenditure; WBCU: whole-body calorimetry unit.

The experimental protocol is illustrated in Figure 1. Potential participants attended a screening visit at the Human Nutrition Research Unit (HNRU) that included the assessment of their height, weight, waist circumference, blood tests (albumin, creatinine, aspartate transaminase, alanine transaminase, sodium, potassium, chloride, and thyroid-stimulating hormone), and the completion of questionnaires eliciting information about health, use of medications, caffeine consumption, physical activity levels [26], and palatability of the study foods. Once deemed eligible, participants were randomly assigned to start with an HP-MR or CON breakfast.

Following a simple randomization procedure separated by sex, participants attended two study visits for the assessment of body composition (GE Lunar iDXA, General Electric Company, Madison, USA; enCORE software 13.60 Lunar iDXA GE Health Care^®^) and resting energy expenditure (REE). Participants also performed a standardized fitness test. Subsequently, participants underwent a 3-day run-in period consuming a eucaloric diet, which preceded both dietary interventions. The day after the run-in period, they received the intervention breakfasts and performed the exercise session while inside the whole-body calorimetry unit (WBCU). Both intervention phases happened during the follicular phase of women’s menstrual cycle. Each intervention phase was followed by a wash-out period of approximately one month for females and two weeks for males.

### 2.3. Resting Energy Expenditure

At baseline, participants completed a 1-h REE indirect calorimetry test, where the volume of oxygen (VO_2_) and carbon dioxide (VCO_2_) were continuously measured by an open-circuit WBCU, a method fully described elsewhere [24]. Results from this test were used to estimate participant’s total energy expenditure (TEE) for the 3-day run-in diet and intervention breakfasts by using the following formula:

Estimated TEE (kcal/day) = REE (kcal/day) × PA × 1.075(1)

where REE was multiplied by a physical activity coefficient (PA), according to the Dietary Reference Intakes [27], and a coefficient of 1.075 representing the metabolizable energy content of the diet [28].

### 2.4. Standardized Fitness Test

At baseline, a fitness test was performed to personalize and standardize the intensity of the prescribed exercise session for each intervention phase. After an 8 to 12 h overnight fast, participants attended the HNRU and were fed a breakfast at 9:00 am with the same energy content and macronutrient composition of the CON breakfast (55% of total energy intake from carbohydrate, 15% from protein, and 30% from fat). At 10:20 am, they performed an incremental submaximal exercise test on a treadmill (Freemotion Incline Trainer, Freemotion Fitness, Logan, UT, USA). The fitness test started with a 5-min warm-up phase in which participants chose a walking speed characterized as comfortable and sustainable. After the warm-up, the incline was increased by 2% every 3 min, and a constant speed was maintained until a respiratory exchange ratio (RER) of 0.90 was achieved. During the test, participant’s heart rate was continuously monitored with a Polar FT1 Heart Rate Monitor (Polar Electro Oy, Kempele, Finland), and expired gases were analyzed by a calibrated TrueMax^®^ metabolic measurement system (Parvo Medics TrueOne^®^ 2400 Metabolic Measurement System, Sandy, UT, USA). The workload for the WBCU exercise session was determined by plotting participant’s RER against their fitness test workload. The speed and incline at which an RER of 0.85 occurred were selected as the intensity for the WBCU exercise sessions.

### 2.5. Run-in Period

Prior to the intervention visits, participants received a 3-day eucaloric diet and were instructed not to eat any other food item, not to consume any caffeinated food products, and to abstain from strenuous exercise. Participants received three meals (breakfast, lunch, and dinner) and two snacks (afternoon and evening snacks) per day that provided 55% of carbohydrate, 15% of protein, and 30% of fat, a macronutrient distribution similar to the CON intervention, which resembled the North American dietary pattern [29].

### 2.6. Energy Metabolism

The morning following the 3-day run-in periods, participants returned to the HNRU after an 8 to 12 h overnight fast, entered the WBCU at 8:00 a.m., received the HP-MR or CON breakfasts at 9:00 a.m., and performed the exercise session from 10:20 a.m. to 11:00 a.m. Energy expenditure, carbohydrate, and fat oxidation rates during the exercise session were calculated from the measurements of VO_2_ and VCO_2_ by using the formula of Brouwer [30]:

EE (kcal) = 3.866 x VO_2_ (L) + 1.20 × VCO_2_ (L) (2)

Carbohydrate Oxidation (g) = 4.170 × VCO_2_ (L) − 2.965 × VO_2_ (L) (3)

Fat Oxidation (g) = 1.718 × VO_2_ (L) − 1.718 × VCO_2_ (L) (4)

Respiratory exchange ratio was calculated as the average ratio of VCO_2_ to VO_2_ per minute during the exercise session:

RER = (VCO_2_ (L))/(VO_2_ (L)) (5)

### 2.7. Interventions

While inside the WBCU, participants received the HP-MR or CON breakfasts at 9:00 a.m. in a random order. The CON breakfast was comprised of whole wheat bread, peanut butter, and orange juice, mimicking the food items and macronutrient distribution of a typical North American breakfast [29]. As an exception, low-fat mozzarella and boiled egg were added to the diets of *n* = 2 participants (females) to increase the energy content of their meals, as their energy requirements were elevated compared to other participants. The HP-MR breakfast consisted of a soy-protein nutritional supplement (Almased^®^, Almased USA, Inc., St. Petersburg, FL, USA) mixed with olive oil and low-fat milk (1% fat), per label instructions [31]. The energy content of the HP-MR and CON breakfasts were similar and represented approximately 20% of participant’s estimated TEE. The nutrient content of the isocaloric breakfasts is described in Table 1. At 10:20 a.m., a 40-min moderate exercise session on a treadmill (BH Fitness T8 SPORT, BH Fitness, Foothill Ranch, CA, USA) was completed, at a personalized fixed pace based on the fitness test’s results.

### 2.8. Appetite Sensations

Throughout each intervention phase, participants rated their appetite sensations (i.e., hunger, satiety, fullness, and prospective food consumption (PFC)) a total of three times using a validated anchored 100-mm visual analogue scale (VAS) [32]: (1) immediately before breakfast (~9:00 a.m.); (2) 30 min after breakfast was finished (~9:40 a.m.); and (3) 1 h after the exercise session (~12:00 p.m.), Figure 1b. The composite satiety score (CSS) was calculated at each time of measurement by using the following equation: CSS (mm) = (satiety + fullness + (100 − prospective food consumption) + (100 − hunger))/4 [33]. A higher CSS is associated with a higher satiety sensation and a subsequent lower motivation to eat.

### 2.9. Metabolic Blood Markers

Blood was sampled by venipuncture at two time points during each intervention phase: 1) in the morning before breakfast (fasting, ~7:30 a.m.); and 2) immediately after the exercise session (post-exercise, ~11:00 a.m.), Figure 1b. The first blood draw was sampled from participants after a 10- to 12-h overnight fast.

Serum samples were analyzed for glucose, insulin, and lipid panel (total cholesterol, high-density lipoprotein (HDL) cholesterol, low-density lipoprotein (LDL) cholesterol, triglyceride, and non-HDL cholesterol) by DynaLIFE Medical Labs (Edmonton, AB, Canada) and leptin in-house at the HNRU. Plasma samples of free fatty acids, non-esterified fatty acids (NEFA), ghrelin (active), PYY, and GLP-1 (active) were analyzed at the HNRU. Leptin and GLP-1 were measured by electrochemiluminescence using the MULTI-ARRAY^®^ Assay System (Meso Scale Discovery^®^, Gaithersburg, MD, USA) and V-PLEX^®^ (Meso Scale Discovery^®^, Gaithersburg, MD, USA), respectively. Ghrelin and PYY were measured by enzyme-linked immunosorbent assay kits from EMD Millipore Co. (Billerica, MA, USA). These analyses were performed according to manufacturer’s instructions, in duplicates, and were repeated when they had not fallen within the range of the standard curves. In females (HP-MR: *n* = 4; CON: *n* = 2), GLP-1 was not detectable in any of the time points and assigned a value of 0.01 pM, defined as the lower limit of detection of the kit. The coefficients of variation (CV) from the serum samples analyzed by DynaLIFE Medical Labs were 1% for glucose, 5% for insulin, 2% for total, HDL, LDL, and non-HDL cholesterol, and 3% for triglyceride. The CVs from the samples analyzed at the HNRU in females and males were 6.26% and 9.18% for NEFA, 7.44% and 7.44% for glycerol, 3.67% and 6.99% for leptin, 6.10% and 5.82% for ghrelin, 7.48% and 10.30% for PYY, and 5.34% and 5.24% for GLP-1, respectively.

### 2.10. Statistical Analysis

Sample size calculation for the primary study has been described elsewhere [24]. Data were expressed as mean ± standard deviation (SD) for continuous variables and frequency and proportions for categorical variables. Paired-samples t-tests were used to compare the mean differences of dietary intake between groups. If dietary intake was nonnormally distributed, Wilcoxon signed-rank tests were used to compare the means between groups. Possible differences between the HP-MR and CON conditions were explored using a mixed analysis of variance (ANOVA) with within-subject factors (i.e., dietary interventions and/or time) and a between-subject factor (i.e., sex). Post-hoc analyses were applied with all ANOVA tests using a Tukey test (equal variances assumed) or Games-Howell (equal variances not assumed). Mean ± standard error of the mean difference (SEM) was used to report the main effect of diet and sex. Partial correlation analyses controlling for sex were performed between continuous variables. IBM^®^ SPSS^®^ Statistics version 24 (International Business Machines Corporation, New York, NJ, USA) was used to perform all statistical analyses. Differences were regarded as statistically significant if *p* < 0.05.

## 3. Results

### 3.1. Participants

**Figure 2:**
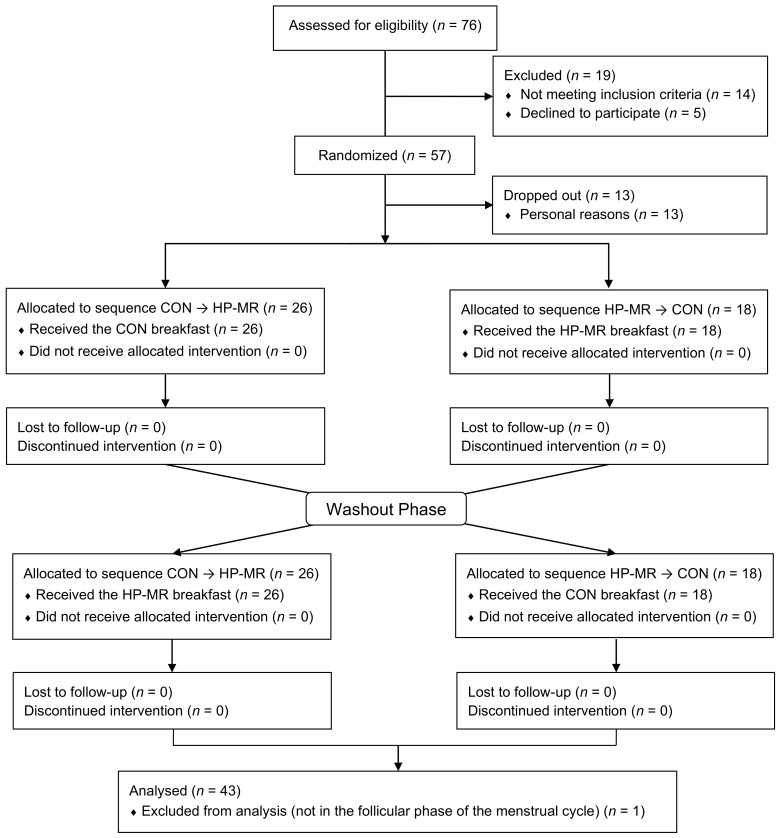
CONSORT flow diagram. CON, control diet; CONSORT, Consolidated Standards of Reporting Trials; HP-MR, high-protein meal replacement. Adapted from Oliveira, Boulé, Sharma, Elliott, Siervo, Ghosh, Berg, and Prado [25].

A total of 76 potential participants were initially screened. After completion of the screening visit, 57 were deemed eligible and randomly assigned to begin with the HP-MR or CON condition. Of these, 13 dropped out before the beginning of the dietary interventions due to personal reasons. Forty-four participants completed the study (*n* = 20 females and *n* = 24 males). One participant (*n* = 1 female) was excluded from data analysis because she was not in the follicular phase of the menstrual cycle during one of the intervention phases, Figure 2. Baseline characteristics of participants are described in Table 2. Seventy-nine percent of participants were classified as active, 16% as moderately active, and 5% as insufficiently active.

### 3.2. Energy Metabolism

**Figure 3:**
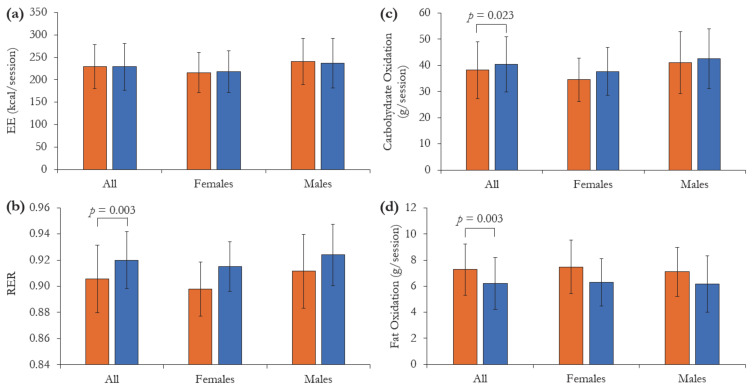
(**a**) Energy expenditure (EE), (**b**) respiratory exchange ratio (RER), (**c**) carbohydrate oxidation and (**d**) fat oxidation, during the exercise session following the consumption of the isocaloric high-protein meal replacement (HP-MR) and control (CON) breakfasts while participants were inside the whole-body calorimetry unit. Values are mean ± standard deviation. *n* = 43 (females *n* = 19; males *n* = 24). *p*-values represent significant difference between the HP-MR and CON groups, as assessed by mixed analysis of variance.

Differences of selected energy metabolism components between the HP-MR and CON conditions are shown in Figure 3. Compared to the CON breakfast, after receiving the HP-MR participants experienced higher fat oxidation rate during the exercise session (1.07 ± 0.33 g/session; *p* = 0.003) and lower carbohydrate oxidation rate (−2.32 ± 0.98 g/session; *p* = 0.023) and RER (−0.01 ± 0.00; *p* = 0.003). Energy expenditure during the exercise did not differ between intervention breakfasts (*p* = 0.833). Although no diet *×* sex interactions were observed in any of the variables assessed (*p* > 0.262), a borderline main effect of sex on RER was detected, in which females presented lower RER than males during the exercise session (−0.01 ± 0.01, *p* = 0.050).

### 3.3. Appetite Sensations

**Figure 4:**
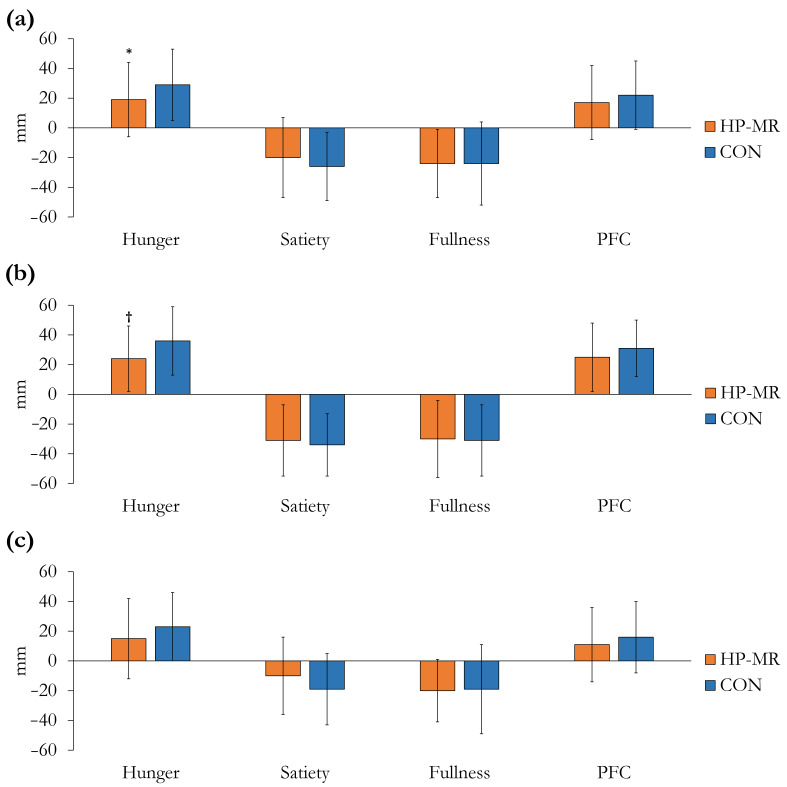
Changes in appetite sensations from after breakfast to after the exercise session during the HP-MR and CON intervention phases in (**a**) all participants (*n* = 43), (**b**) females (*n* = 19), and (**c**) males (*n* = 24). Values are mean ± standard deviation. * Significant difference (*p* = 0.014) in all participants (*n* = 43) between the HP-MR and CON interventions, as assessed by mixed analysis of variance. ^†^ Significant difference (*p* = 0.019) in females (*n* = 19) between the HP-MR and CON interventions, as assessed by mixed analysis of variance. Abbreviations: CON: control, standard North American diet; HP-MR: high-protein meal replacement; PFC: prospective food consumption.

Participant’s appetite sensations before breakfast were not different between the HP-MR and CON conditions, except from PFC, which was lower in the HP-MR compared to the CON (−5 ± 15 mm; *p* = 0.045). Changes in appetite sensations from after breakfast to after the exercise session are illustrated in Figure 4. Compared to the CON intervention, hunger increased less in the HP-MR condition (−10 ± 4 mm; *p* = 0.014), Figure 4a. The change in satiety (*p* = 0.229), fullness (*p* = 0.955), and PFC (*p* = 0.218) was not different between interventions. No interaction between dietary interventions *×* sex was observed in any of the appetite sensations (*p* ≥ 0.484). In females, the increase in hunger was lower in the HP-MR compared to the CON condition (−12 ± 5 mm; *p* = 0.019), Figure 4b.

**Figure 5:**
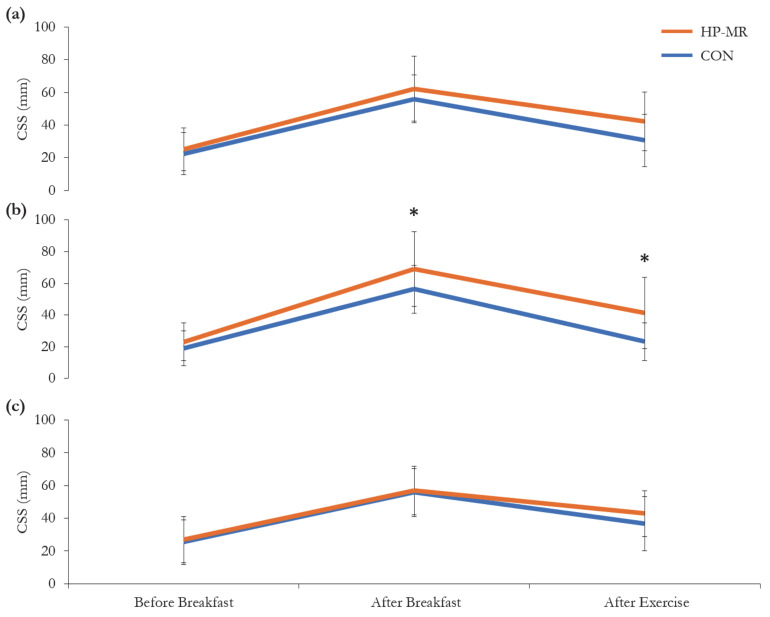
Composite satiety score during the HP-MR and CON interventions in (**a**) all participants (*n* = 43), (**b**) females (*n* = 19), and (**c**) males (*n* = 24). Data are mean ± standard deviation. * Significant difference (*p* < 0.03) between the HP-MR and CON diets, as assessed by a mixed analysis of variance. Abbreviations: CON: control, standard North American diet; CSS: composite satiety score; HP-MR: high-protein meal replacement.

No significant three-way interaction between diet, sex and time for CSS (*p* = 0.322) was observed; however, there was a significant two-way interaction between diet and sex (*p* = 0.037), and diet and time for females (*p* = 0.036), but not for males (*p* = 0.489), Figure 5. In females, CSS was higher after breakfast and after the exercise session in the HP-MR compared to the CON condition (13 ± 5 mm; *p* = 0.034; 18 ± 5 mm; *p* = 0.003, respectively), while no difference was observed in males in any of the time points (*p* > 0.05). In the HP-MR condition, CSS after the exercise session was higher in females compared to males (12 ± 6 mm; *p* = 0.048), while in the CON group, CSS after the exercise session was significantly lower in females compared to males (−14 ± 4 mm; *p* = 0.005).

### 3.4. Metabolic Blood Markers

Metabolic blood markers assessed in a fasting state and after the exercise session during the HP-MR and CON conditions are shown in Table 3. Compared to the CON intervention, the change in blood markers from the fasting state to post-exercise in the HP-MR was greater for insulin (19.2 ± 9.1 pmol/L; *p* = 0.042), LDL cholesterol (0.08 ± 0.02 mmol/L; *p* = 0.003), PYY (22.78 ± 10.19 pg/mL; *p* = 0.031), and GLP-1 (1.45 ± 0.40 pM; *p* = 0.001), and lesser for triglyceride (−0.14 ± 0.04 mmol/L; *p* = 0.002), and glycerol (−6.4 ± 2.5 µM; *p* = 0.015). On the other hand, this change was not different between the dietary interventions for glucose, total cholesterol, HDL cholesterol, non-HDL cholesterol, NEFA, leptin, and ghrelin, *p* > 0.05.

There was a significant interaction between diet and sex on the change from the fasting state to post-exercise in GLP-1 concentration (*p* = 0.009). In males, the change in GLP-1 was greater in the HP-MR compared to the CON condition (2.55 ± 0.65 pM; *p* = 0.001), while in females, this change was not different between dietary interventions (0.35 ± 0.30 pM; *p* = 0.252). In both dietary intervention groups, the change in GLP-1 was greater in males compared to females (HP-MR: 5.04 ± 0.64 pM; *p* < 0.001; CON: 2.80 ± 0.74 pM; *p* = 0.001).

## 4. Discussion

The primary findings of our study were that, compared to a standard North American meal (CON), the HP-MR led to higher fat oxidation during the exercise session, and a suppression of hunger and improved metabolic profile after exercise. Females and males responded differently to the dietary interventions. Females presented a stronger response in appetite sensations, while in males, this response was related to the appetite-related hormone GLP-1. Interestingly, all these effects were produced with an acute nutritional intervention and in the absence of a difference in exercise EE between groups. These results highlight the impact an HP-MR has during and after an exercise session on energy metabolism, appetite sensations, and metabolic blood markers of healthy adults, and provides further insight into the potential role of these combined strategies for weight management.

This study showed that consumption of the HP-MR led to higher fat oxidation and lower carbohydrate oxidation during the exercise session, which is reflected by lower RER levels observed following the consumption of this dietary intervention. It is well known that substrate oxidation during exercise is highly influenced by substrate availability [34], meaning that dietary intake is an important determinant of nutrient partitioning during exercise. The HP-MR breakfast had carbohydrate levels below the Acceptable Macronutrient Distribution Range (i.e., ~30% of total energy intake), which characterizes this dietary intervention as low-carbohydrate [27]. It has been demonstrated that carbohydrate consumption directly regulates fat oxidation at rest [35] and during exercise [34], although the exact mechanisms behind this regulation remain to be fully understood [36]. These observations are in agreement with the results presented herein and are further supported by another study that observed increased fat oxidation during and after a moderate-intensity aerobic exercise following the consumption of an acute low-carbohydrate diet in healthy, normal-weight women [37]. Although the difference in substrate oxidation rates and RER values between interventions was statistically significant, the clinical meaningfulness of the relatively small numbers is unknown. However, even small changes in nutrient partitioning towards increased fat oxidation from dietary manipulation and physical activity result in significant changes in body weight and composition over the long term [8,38,39]. In fact, low RER values, and hence low rates of fat oxidation, have been shown to predict long-term weight gain [40,41].

The potential compensatory increases in energy intake in response to an exercise-induced energy deficit have been discussed since the 1950s [42,43]. Although the exact mechanisms underlying the causes of this compensation are still poorly understood, more recent evidence supports the hypothesis that an increase in hunger and energy intake is due to an increase in EE resultant from exercise practice [44,45]. This compensation can undermine the exercise-induced weight-loss, which partially explains why some individuals do not lose or even gain body weight after starting an exercise training program [44]. Therefore, dietary interventions able to minimize this compensation have the potential to improve an individual’s response to exercise-induced energy deficit and, consequently, weight loss. In this study, the increase in hunger was lower after the exercise session when individuals consumed the HP-MR compared to the CON breakfast. This effect might be related to the higher protein content of the HP-MR. Protein is the most satiating macronutrient, followed by carbohydrate and fat [22]. Different mechanisms and pathways seem to be involved in the appetite responses to dietary protein, such as secretion of gut hormones, effects on digestion, blood concentrations of amino acids, and EE [46]. In a randomized, cross-over trial, Dougkas and Östman [47] fed young adults isovolumetric and isoenergetic liquid meals matched for energy density and sensory properties with increasing amounts of dietary protein (i.e., 9%, 24%, and 40% of total energy intake). They reported that most appetite ratings were suppressed with increasing protein content of the test meals [47], which is in line with our study’s findings. Interestingly, even though the HP-MR was in liquid form and the CON breakfast was solid, the HP-MR was still able to suppress hunger to a greater extent after the exercise session. Research has shown that the physical state of the food can affect appetite sensations [48]. When comparing a high-protein solid meal versus a liquid one of identical nutrient profile, researchers observed that the solid version was able to evoke stronger suppression of hunger and desire to eat than the liquid meal in healthy, normal-weight, young adults [49]. Therefore, according to our study’s results, it seems that the macronutrient distribution of the meal might have a stronger effect on hunger suppression than the physical state of it.

After the exercise session, the increase in GLP-1 and PYY was higher with the consumption of the HP-MR compared to the CON breakfast, confirming the findings discussed above on hunger suppression. These two anorexigenic hormones are synthesized and released from the L-cells of the gastrointestinal tract and have been shown to modulate functional brain activation after food intake decreasing hunger and promoting meal cessation [50,51]. Both gut-derived peptides are secreted in response to nutrient intake, particularly dietary protein [52]. In a similar study design, Lejeune, et al. [53] fed healthy, normal-weight women an energy-balanced diet comprised of 10% or 40% of protein for 36 h while participants stayed inside a WBCU. The authors demonstrated that blood levels of GLP-1 were significantly higher after dinner when participants were fed the diet comprised of 40% protein [53]. Similar to the effects on GLP-1, research has shown that a high-protein meal increases PYY in normal-weight individuals to a greater extent than a high-carbohydrate or high-fat meal [54]. Therefore, the higher protein content of the HP-MR breakfast seems to be the biggest contributor to the greater increase in GLP-1 and PYY observed in this study. In addition to the effects observed on the secretion of gut-derived peptides, the HP-MR breakfast also increased blood insulin levels to a greater extent after the exercise session than the CON breakfast. This effect seems to be related to the type of dietary protein found in the HP-MR (i.e., soy). It has been shown that the consumption of a high-protein meal containing soy increased insulin secretion in both primates [55] and humans [56,57]. This effect seems to be related to the protein content of the meal [57] and the isoflavone genistein contained in the soy, which seems to exert an insulinotropic effect by acting directly on pancreatic β-cells [58].

In our study, triglyceride blood concentration increased less from the fasting state to post-exercise after participants ingested the HP-MR compared to the CON break-fast. The low carbohydrate content of this dietary intervention might have been re-sponsible for this effect [59]. This was demonstrated by Wolfe and Piche [60], who ob-served a reduction in blood triglyceride levels of healthy individuals after replacing dietary carbohydrate with protein in a diet with fixed fat content. On the other hand, blood concentration of LDL cholesterol decreased less from the fasting state to post-exercise after participants ingested the HP-MR compared to the CON breakfast. This effect might be partially related to the lower dietary fibre of the HP-MR. Dietary fibre is known for its cholesterol-lowering effects [61], as it binds to bile acids in the in-testinal lumen decreasing cholesterol reabsorption during the enterohepatic cycle [62]. Additionally, blood glycerol concentration increased less from the fasting state to post-exercise after participants ingested the HP-MR compared to the CON breakfast. Circulating glycerol results mainly from hydrolysis of triglyceride stored in adipose tissue and constitutes a major substrate for glucose homeostasis [63]. The increased fat oxidation observed during exercise after the consumption of the HP-MR suggests an increased hydrolysis of triglyceride in adipose tissue and subsequent use of circulating glycerol as an energy source, which might have contributed to its reduced circulating levels.

Interestingly, the improved metabolic profile associated with the ingestion of the HP-MR occurred in the context of no differences in exercise EE between groups. Manore, Larson-Meyer, Lindsay, Hongu, and Houtkooper [8] discuss that exercise affects energy balance beyond simply expending energy. In fact, the most recent Canadian Adult Obesity Clinical Practice Guidelines states that regular physical activity should be part of weight management interventions, as it produces several health benefits, even in the absence of weight loss [64]. For instance, Barwell, Malkova, Leggate, and Gill [39] reported that the shift in fasting RER resulted from exercise training was independently associated with the change in fat mass in adult females. Moreover, increased fat oxidation has been shown to result in metabolic benefits beyond the regulation of body weight, such as improvement in insulin sensitivity [65,66]. In addition, the HP-MR decreased hunger and increased blood levels of the anorexigenic hormones GLP-1 and PYY after the exercise session, which can ultimately affect energy intake and hence body weight regulation. Altogether, the improved metabolic profile associated with the ingestion of the HP-MR prior to the exercise session can ultimately potentially affect the energy balance equation and produce several health-related benefits, even in the absence of an increase in exercise EE.

In this study, the HP-MR breakfast elicited different responses in females and males in respect to appetite sensations and its related hormones. In females, hunger was affected, while in males, the effect was only on GLP-1 blood levels, their appetite sensations were not impacted. These results suggest that female’s appetite sensations were more sensitive and reactive to dietary manipulation than male’s. Our results are in agreement with a previously conducted randomized, cross-over study testing an acute dietary intervention comprised of 10% or 30% protein in healthy adults of both sexes [67]. Differences in hunger and satiety between the diets were more pronounced in females than in males, while the increase in GLP-1 was greater in males with the higher protein diet [67], which is in agreement with our study results. The authors discussed that weight-loss strategies should be different by sex and that diets able to stimulate satiety should be preferred as a weight loss strategy for females. Differences between sexes in gonadal steroid hormones [68] and neuronal responses to food intake [69] partly explain the discrepancy in appetite sensations observed herein. Estrogen has been shown to inhibit food intake, and this hormone is higher during the follicular phase of the menstrual cycle [70,71], the cycle phase of females tested in our study. Although GLP-1 blood levels increased more in males than in females, it has been demonstrated that its release is lower during the follicular phase of the menstrual cycle compared to the luteal phase due to the lower levels of estrogen and progesterone, two hormones that exert important effects on appetite-related hormones [70,72]. Considering that females were on the follicular phase of their menstrual cycle, the effect of this dietary intervention in the luteal phase remains to be elucidated.

Strengths of this study include a well-controlled study design and dietary intervention. Moreover, state-of-the-art techniques for the assessment of exercise energy metabolism were used. However, some limitations must be acknowledged, such as the acute intervention, specificity of the population group (i.e., healthy, normal-weight young adults), a selective menstrual cycle phase, and the fact that the HP-MR and CON conditions were not performed without exercise. Moreover, this study had a limited number of assessments of appetite sensations and appetite-related hormones. These limitations restrict our ability to translate these results to longer intervention periods, other population groups, to other phases of the female menstrual cycle and hamper our ability to conclude the exact role of exercise on the findings. Therefore, future studies are needed to better understand the long-term effects of this intervention on the physiology of healthy and diseased population groups in more than one phase of female’s menstrual cycle. Moreover, feeding participants the HP-MR and CON breakfasts without exercise would increase our ability and understand the real effects of exercise on the findings presented herein.

## 5. Conclusions

In conclusion, this study showed that, compared to a standard North American breakfast (CON), an isocaloric HP-MR led to higher fat oxidation during exercise, suppression of hunger, and improved metabolic profile after exercise. Females and males responded differently to the dietary interventions. Females presented a stronger response in appetite sensations, while in males, this response was related to the appetite-related hormone GLP-1. These results highlight the impact HP-MR consumption has during and after an exercise session on energy metabolism, appetite sensations, and metabolic blood markers of healthy, normal-weight adults of both sexes, and provides further insight into the potential role of these combined strategies for weight management.

## Figures and Tables

**Table 1:** Nutrient content of the intervention breakfasts.

	HP-MR			CON			Diet Difference *^a^*
	All (*n* = 43)	Females (*n* = 19)	Males (*n* = 24)	All (*n* = 43)	Females (*n* = 19)	Males (*n* = 24)	
Energy							
Kcal/meal	413 ± 74	366 ± 59	450 ± 63	409 ± 72	360 ± 51	448 ± 63	<0.001
kcal/kg body weight	6 ± 1	6 ± 1	7 ± 1	6 ± 1	6 ± 1	7 ± 1	<0.001
Protein							
% energy	42.6 ± 0.8	43.0 ± 0.9	42.3 ± 0.4	14.7 ± 0.8	14.3 ± 1.1	15.1 ± 0.2	<0.001
g/meal	44 ± 7	39 ± 6	47 ± 6	16 ± 3	14 ± 3	17 ± 2	<0.001
g/kg body weight	0.7 ± 0.1	0.6 ± 0.1	0.7 ± 0.1	0.2 ± 0.0	0.2 ± 0.0	0.3 ± 0.0	<0.001
Fat							
% energy	26.6 ± 0.6	26.4 ± 0.4	26.7 ± 0.6	30.2 ± 1.8	30.5 ± 2.8	29.9 ± 0.3	<0.001
g/meal	12 ± 2	11 ± 2	13 ± 2	14 ± 3	12 ± 2	15 ± 2	<0.001
g/kg body weight	0.2 ± 0.0	0.2 ± 0.0	0.2 ± 0.0	0.2 ± 0.0	0.2 ± 0.0	0.2 ± 0.0	<0.001
Carbohydrate							
% energy	30.8 ± 0.6	30.6 ± 0.8	31.0 ± 0.4	55.0 ± 1.9	55.2 ± 2.8	54.9 ± 0.3	<0.001
g/meal	32 ± 6	28 ± 5	35 ± 5	58 ± 10	52 ± 8	63 ± 9	<0.001
g/kg body weight	0.5 ± 0.1	0.4 ± 0.1	0.5 ± 0.1	0.9 ± 0.1	0.8 ± 0.1	0.9 ± 0.1	<0.001
Sugars (g/meal)	32 ± 6	28 ± 5	35 ± 5	27 ± 4	25 ± 3	29 ± 4	<0.001
Fiber (g/meal)	1 ± 0	1 ± 0	1 ± 0	7 ± 1	6 ± 1	8 ± 1	<0.001
Saturated Fat (g/meal)	3 ± 0	2 ± 0	3 ± 0	3 ± 1	3 ± 0	3 ± 0	<0.001
Monounsaturated Fat (g/meal)	7 ± 1	6 ± 1	8 ± 1	6 ± 1	6 ± 1	7 ± 1	<0.001
Polyunsaturated Fat (g/meal)	1 ± 0	1 ± 0	1 ± 0	4 ± 1	3 ± 1	4 ± 1	<0.001
Cholesterol (mg/meal)	12 ± 3	10 ± 3	13 ± 2	4 ± 28	10 ± 42	0 ± 0	<0.001

Data are expressed as mean ± standard deviation. *^a^* *p*-values represent the difference between groups in all participants (*n* = 43) and were detected with the use of paired-samples t-test or Wilcoxon signed-rank test, accordingly. Abbreviations: CON: control, standard North American diet; HP-MR: high-protein meal replacement.

**Table 2:** Baseline characteristics of participants.

Characteristics	All (*n* = 43)
Age (years)	24 ± 4
Height (cm)	171.1 ± 7.3
Weight (kg)	64.4 ± 6.9
Waist Circumference (cm)	74.4 ±5.6
BMI (kg/m^2^)	22.0 ± 1.4
FM (kg) (F/M)	18.6 ± 3.3/12.7 ± 4.9
LST (kg) (F/M)	40.1 ± 4.4/51.4 ± 5.6
Ethnicity	
White	19 (44)
Asian	14 (33)
Hispanic	3 (7)
Black	1 (2)
Other	6 (14)

Data are expressed as mean ± standard deviation or *n* (%). Abbreviations: BMI: body mass index; F: females; FM: fat mass; LST: lean soft tissue; M: males.

**Table 3:** Metabolic blood markers before and after the exercise session.

	HP-MR					CON				∆*^a^*		
	Fasting	Post-Exercise	∆ *^a^*	Time Effect *^b^*	Time *×* Sex *^b^*	Fasting	Post-Exercise	∆ *^a^*	Time Effect *^b^*	Time *×* Sex *^b^*	Diet Effect *^c^*	Diet *×* Sex *^c^*
Glucose (mmol/L)	4.8 ± 0.3	5.1 ± 0.4	0.3 ± 0.4	<0.001	0.371	4.8 ± 0.3	5.1 ± 0.6	0.2 ± 0.6	0.008	0.366	0.662	0.800
Insulin (pmol/L) *^d^*	43.1 ± 15.4	95.8 ± 59.7	52.9 ± 52.2	<0.001	0.123	44.3 ± 18.4	78.5 ± 40.0	34.1 ± 33.2	<0.001	0.057	0.042	0.744
Lipid Panel *^d^*												
Total Cholesterol (mmol/L)	4.34 ± 0.73	4.37 ± 0.73	0.02 ± 0.18	0.196	0.017	4.28 ± 0.68	4.29 ± 0.69	0.01 ± 0.17	0.643	0.294	0.528	0.266
HDL Cholesterol (mmol/L)	1.45 ± 0.43	1.46 ± 0.45	0.01 ± 0.06	0.125	0.056	1.43 ± 0.43	1.44 ± 0.43	0.01 ± 0.07	0.214	0.795	0.987	0.251
Non-HDL Cholesterol (mmol/L)	2.89 ± 0.57	2.90 ± 0.55	0.01 ± 0.13	0.287	0.020	2.85 ± 0.49	2.85 ± 0.49	−0.01 ± 0.11	0.909	0.146	0.323	0.376
LDL Cholesterol (mmol/L)	2.40 ± 0.52	2.38 ± 0.48	−0.02 ± 0.13	0.314	0.010	2.36 ± 0.48	2.26 ± 0.47	−0.11 ± 0.14	<0.001	0.014	0.003	0.978
Triglyceride (mmol/L)	1.06 ± 0.42	1.15 ± 0.50	0.08 ± 0.19	0.008	0.829	1.07 ± 0.42	1.30 ± 0.51	0.22 ± 0.27	<0.001	0.194	0.002	0.337
Glycerol (µM) *^e^*	27.5 ± 19.6	36.3 ± 28.8	7.8 ± 15.1	<0.001	0.001	23.1 ± 14.1	37.3 ± 22.3	14.3 ± 14.8	<0.001	0.001	0.015	0.975
NEFA (µM) *^e^*	201.2 ± 191.6	188.5 ± 222.9	−13.9 ± 204.4	0.774	0.296	176.5 ± 147.9	183.6 ± 173.4	7.1 ± 114.9	0.607	0.391	0.514	0.551
Leptin (pg/mL) *^d^*	8702.86 ± 9994.55	7418.60 ± 8780.62	−1241.71 ± 1903.88	<0.001	<0.001	9026.12 ± 10798.76	7300.37 ± 8360.41	−1725.74 ± 3147.62	<0.001	<0.001	0.154	0.153
PYY (pg/mL) *^f^*	123.34 ± 61.70	196.76 ± 79.42	73.90 ± 50.70	<0.001	0.265	124.89 ± 64.05	168.55 ± 79.87	47.65 ± 61.48	<0.001	0.229	0.031	0.052
GLP-1 (pM) *^e^*	1.44 ± 3.17	5.03 ± 5.59	3.68 ± 3.25	<0.001	<0.001	1.47 ± 3.16	3.54 ± 4.47	2.07 ± 2.74	<0.001	0.001	0.001	0.009
Ghrelin (pg/mL) *^e^*	442.76 ± 296.13	292.54 ± 190.23	−149.38 ± 157.07	<0.001	0.023	474.33 ± 316.86	288.58 ± 183.35	−185.75 ± 199.17	<0.001	0.009	0.128	0.335

Data are presented as mean ± standard deviation. *n* = 43, unless otherwise stated. *^a^* ∆: Post-exercise minus baseline values. *^b^* *p*-values represent the effect of time within groups and were detected with the use of a mixed analysis of variance. *^c^* *p*-values represent the effect of the dietary interventions on the ∆ (i.e., change from baseline to post-exercise) between groups and were detected with the use of a mixed analysis of variance. *^d^* HP-MR: *n* = 42; CON: *n* = 43. *^e^* HP-MR: *n* = 43; CON: *n* = 42. *^f^* HP-MR: *n* = 42; CON: *n* = 41. Abbreviations: CON: control, standard North American diet; GLP-1: Glucagon-like peptide; HDL: high-density lipoprotein; HP-MR: high-protein meal replacement; LDL: low-density lipoprotein; NEFA: non-esterified free fatty acids; PYY: Peptide YY.

## Data Availability

The data presented in this study are available upon request from the corresponding authors.
